# Highly Luminescent and Photostable Core/Shell/Shell ZnSeS/Cu:ZnS/ZnS Quantum Dots Prepared via a Mild Aqueous Route

**DOI:** 10.3390/nano12183254

**Published:** 2022-09-19

**Authors:** Salima Mabrouk, Hervé Rinnert, Lavinia Balan, Jordane Jasniewski, Sébastien Blanchard, Ghouti Medjahdi, Rafik Ben Chaabane, Raphaël Schneider

**Affiliations:** 1 Université de Lorraine, CNRS, LRGP, F-54000 Nancy, France; 2 Laboratoire Interfaces et Matériaux Avancés, LIMA, LR011ES55, Faculté des Sciences de Monastir, Avenue de l’Environnement, Monastir 5019, Tunisia; 3 Université de Lorraine, CNRS, IJL, F-54000 Nancy, France; 4 CEMHTI-UPR 3079 CNRS, Site Haute Température, 1D Avenue de la Recherche Scientifique, 45071 Orléans, France; 5 Université de Lorraine, LIBio, F-54000 Nancy, France; 6 Sorbonne Université, CNRS, Institut Parisien de Chimie Moléculaire, IPCM, F-75005 Paris, France

**Keywords:** core/shell/shell ZnSeS/Cu:ZnS/ZnS quantum dots, dopant location, optoelectronic properties, (photo)stability

## Abstract

An aqueous-phase synthesis of 3-mercaptopropionic acid (3-MPA)-capped core/shell/shell ZnSeS/Cu:ZnS/ZnS QDs was developed. The influence of the Cu-dopant location on the photoluminescence (PL) emission intensity was investigated, and the results show that the introduction of the Cu dopant in the first ZnS shell leads to QDs exhibiting the highest PL quantum yield (25%). The influence of the Cu-loading in the dots on the PL emission was also studied, and a shift from blue–green to green was observed with the increase of the Cu doping from 1.25 to 7.5%. ZnSeS/Cu:ZnS/ZnS QDs exhibit an average diameter of 2.1 ± 0.3 nm and are stable for weeks in aqueous solution. Moreover, the dots were found to be photostable under the continuous illumination of an Hg–Xe lamp and in the presence of oxygen, indicating their high potential for applications such as sensing or bio-imaging.

## 1. Introduction

Over the past two decades, semiconductor nanocrystals, also called quantum dots (QDs), have attracted high attention due to their outstanding electronic and optical properties and the associated applications (LEDs, solar cells, lasers, photoluminescent probes, et al.) [1,2,3]. QDs containing heavy metals such as CdSe, CdTe or PbS have been extensively studied because both their absorption and their photoluminescence (PL) emissions can span over the whole visible region and in the near infrared. However, the toxicity of Cd and Pb, listed as Class A elements, severely limits the potential applications of Cd or Pb-based QDs for numerous applications.

Recently, transition metal-doped wide bandgap QDs such as ZnS or ZnSe have gained high interest as alternatives to heavy metal containing QDs not only due to their lower toxicity but also due their new optical properties [4,5,6,7]. Indeed, the doping generates deep trap levels acting as PL centers, which confer to the dots exceptional properties such as a long PL excited state lifetime, high quantum efficiency and large Stokes shift that minimize the self-absorption [4,5,6,7]. Moreover, doped ZnS or ZnSe QDs exhibit higher chemical, photo and thermal stability than conventional Cd- or Pb-based binary QDs [8,9].

Ternary QDs such as ZnSeS offer many advantages compared to binary ones because their electronic and optical properties can be tuned by varying their diameter and their composition but also due to their high stability [10,11,12,13]. The doping of ZnSeS QDs with Mn^2+^ has recently attracted interest, but the PL emission tunability is relatively limited as the exciton photo-generated in the ZnSeS host recombines with the lower lying states of the Mn^2+^ ion (^4^T_1_ → ^6^A_1_ transition), which leads to a characteristic orange emission [13,14,15,16,17]. The use of Cu^2+^ as dopant in ZnSeS QDs has been less investigated despite the interest in these nanocrystals for many applications such as bioimaging or sensing as their PL emission is more color tunable than Mn^2+^-doped QDs [18,19]. Only two reports describe the aqueous-phase synthesis of Cu-doped ZnSeS QDs; in both cases, the Cu dopant is located in the ZnSeS core. De S. Viol et al. reported the preparation of 3-mercaptopropionic (3-MPA)-capped Cu:ZnSe QDs. Due to the partial decomposition of the 3-MPA ligand and the associated release of S^2−^ anions in the course of the synthesis (100 °C for 24 h), S^2−^ ions diffuse into the ZnSe core to produce Cu:ZnSeS QDs emitting at 515 nm [20]. A similar strategy was used by Zeng et al., except that the reaction was conducted in an autoclave at 180 °C, which favors the decomposition of 3-MPA and the formation of the alloyed ZnSeS core more [21]. Noteworthy is also that both synthetic methods do not allow the control of the Se/S atomic ratio in the ZnSeS alloyed core as S^2−^ is produced in situ, and its concentration will depend on the rate of decomposition of the MPA ligand.

A few studies have shown that the location of the Cu dopant within the QDs affects the PL intensity and the PL lifetime of the dopant [22,23,24]. For Cu-doped CdSe QDs prepared in organic phase, the formation of Cu^+^ antisite defects increases with the distance from the QD center, while for Cu^2+^, an opposite trend is found [24]. Thus, Cu^+^ and Cu^2+^ impurities should preferentially be incorporated closer to the QD center and nearer to the QD surface, respectively. For Cu-doped ZnS or ZnSe QDs prepared in aqueous phase using a Cu^2+^ complex and Na_2_S or NaHSe as Cu, S and Se precursors, respectively, the situation is more complex as Cu^2+^ is usually reduced into Cu^+^ during the synthesis by S^2−^ or Se^2−^ ions [19,20,25]. After excitation with UV light, Cu^+^ binds with the hole in the valence band of the ZnS or ZnSe host generating a transient Cu^2+^ acceptor state. Next, the electron promoted into the conduction band recombines with the hole trapped in the Cu^2+^ d state, which leads to light emission and the reformation of Cu^+^.

We recently developed the preparation of 3-MPA-capped Cu-doped core/shell ZnSeS/ZnS and showed their potential as photoluminescent probes for the detection of Pb^2+^ ions [19]. In this paper, a systematic study of the influence of the dopant position, of its concentration and of the thickness of the ZnS shell on the optical properties of Cu-doped core/shell ZnSeS/ZnS QDs is described. Our results show that a high PL quantum yield of 25% could be achieved by doping the first ZnS shell with 2.5% Cu and by depositing two monolayers of ZnS at the periphery of ZnSeS/Cu:ZnS QDs. Moreover, the PL emission could be tuned from 480 to 510 nm by increasing the dopant loading from 0% to 7.5%. Finally, we demonstrate that ZnSeS/Cu:ZnS/ZnS QDs are photostable in the presence of oxygen.

## 2. Materials and Methods

### 2.1. Materials

Zinc nitrate hexahydrate Zn(NO_3_)_2_•6H_2_O (>99.0%, Sigma-Aldrich, Saint-Quentin-Fallavier, France), 3-mercaptopropionic acid (>99%, Sigma-Aldrich, Saint-Quentin-Fallavier, France), selenium powder (99.5+%, Sigma-Aldrich, Saint-Quentin-Fallavier, France), sodium borohydride (99%, Sigma-Aldrich, Saint-Quentin-Fallavier, France), sodium sulfide nonahydrate Na_2_S•9H_2_O (98.0% min, Alfa Aesar, Kandel, Germany), copper(II) acetate monohydrate Cu(OAc)_2_•H_2_O (≥99.0%, Merck, Saint-Quentin-Fallavier, France) and absolute ethanol (VWR) were used without further purification. Deionized Milli-Q water was used as solvent.

### 2.2. Synthesis of Core/Shell/Shell ZnSeS/Cu:ZnS/ZnS QDs

A typical synthesis of ZnSeS/Cu:ZnS/ZnS QDs doped with 2.5% Cu is described: Zn(NO_3_)_2_ (0.75 mmol) and 3-MPA (100 μL, 1.147 mmol) are dissolved in 20 mL of water until a homogeneous solution is obtained. Next, the pH of the solution is adjusted to 11 with 1 M NaOH before being deoxygenated by Ar bubbling for 30 min. Separately, NaHSe is prepared by reacting Se(0) (0.215 mmol) with NaBH_4_ (7.93 mmol) in 4 mL of water. The colorless solution obtained after 10 min of stirring under argon is quickly injected to the Zn(NO_3_)_2_ and 3-MPA solution under continuous stirring for 15 min. Next, Na_2_S•9H_2_O (0.215 mmol) in 2 mL of water is injected, and the solution is mixed for 5 min at room temperature. Finally, the reaction mixture is brought to boiling for 4 h to obtain ZnSeS core QDs.

For the Cu-doped ZnS shell growth on ZnSeS QDs, 0.65 mL of a 0.15 M Zn(NO_3_)_2_ solution containing Cu(OAc)_2_ (0.0187 mmol) and 0.65 mL of a 0.1 M Na_2_S•9H_2_O/0.04 M 3-MPA solution (pH adjusted to 11) were added dropwise using two syringes to the solution containing ZnSeS QDs. After 1 h heating at 100 °C under stirring, a second ZnS shell was deposited via the same method. One injection of Zn^2+^ and S^2−^ precursors allowed the installation of a ZnS monolayer to the surface of ZnSeS cores. After cooling, the final ZnSeS/ZnS:Cu/ZnS QDs were purified by precipitation using ethanol followed by centrifugation (1700× *g* for 15 min). QDs were further washed with ethanol (2 × 20 mL) before being dried in vacuum before characterization.

### 2.3. Photostability Experiments

The photostability of ZnSeS/Cu:ZnS/ZnS QDs was assessed by irradiating for 1 h an aqueous dispersion of the dots with an Hg–Xe lamp (irradiance of 50 mW/cm^2^). The Fluorescein organic dye was used as reference.

### 2.4. Characterization

Transmission electron microscopy (TEM), high-resolution (HR-TEM) images and selected-area electron diffraction (SAED) analyses were conducted on a Philips CM200 instrument (Philips, Suresnes, France) operating at 200 kV.

X-ray diffraction (XRD) measurements were conducted at room temperature using a Panalytical X’Pert Pro MPD (Malvern Panalytical, UK) diffractometer using Cu Kα radiation (λ = 0.15418 nm).

X-ray photoelectron spectroscopy (XPS) was performed using a Gammadata Scienta SES 200-2 spectrometer (Uppsala, Sweden).

A Zetasizer Nano ZS (Malvern Panalytical, UK), equipped with a He/Ne ion green laser (λ = 532 nm), was used in a backscattering configuration (173°) to determine the hydrodynamic diameter, the polydispersity indexes (PDI) and Zeta potentials of QDs.

Thermogravimetric analysis (TGA) was performed under an O_2_ atmosphere from room temperature to 800 °C (heating rate of 10 °C/min) using a TGA/DSC1 STAR equipment (Mettler-Toledo, Viroflay, France).

The Fourier transform infrared (FT-IR) transmittance spectra of QDs were recorded on a Bruker ALPHA spectrometer (Bruker, Palaiseau, France).

UV-visible absorption spectra were obtained using a Thermo Scientific Evolution 220 spectrophotometer (Thermo Fisher, Illkirch-Grafenstaden, France).

PL measurements were performed using a Horiba Fluoromax-4 Jobin Yvon spectrometer (HORIBA Jobin Yvon, Longjumeau, France). PL spectra were corrected, and PL QYs were measured using Fluorescein as a reference standard (PL QY = 95% in a 0.1 M NaOH solution).

The PL decay curves were recorded using a excitation of 10 ns pulses at 355 nm emitted by an yttrium aluminium garnet (YAG):Nd laser. The PL signal was measured by a InGaAs/InP photomultiplier tube. The rise time of the detector was ca. 3 ns.

The Cu dopant concentration of ZnSeS/Cu:ZnS/ZnS QDs after purification and acid digestion was determined by inductively coupled plasma–optical emission spectrometry (ICP-OES, Varian 720-ES equipment, Varian, Le Plessis-Robinson, France).

Electron Spin Resonance (ESR) measurements were carried out at 20 K in non-saturating conditions using a Bruker ELEXSYS 500 spectrometer (Bruker, Palaiseau, France). Typical measurement conditions were a microwave power of 0.63 mW, microwave frequency of 9.409 GHz and modulation amplitude of 5G.

## 3. Results and discussion

### 3.1. Synthesis and Optical Properties of ZnSeS/Cu:ZnS/ZnS QDs

**Scheme 1:**
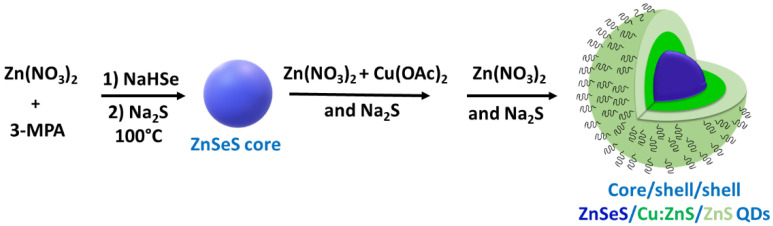
Schematic description of ZnSeS/Cu:ZnS/ZnS QDs synthesis.

ZnSeS/Cu:ZnS/ZnS QDs were prepared in aqueous phase at 100 °C using 3-MPA as capping ligand as described in Scheme 1.

First, the influence of the Se/S ratio on the optical properties of the alloyed ZnSeS core was evaluated. As can been seen in Figure S1, the onset of the absorption shifts to higher wavelengths with the increase of the Se content due to the larger bandgap of ZnS (3.7 eV) compared to ZnSe (2.7 eV). No well-defined excitonic peak could be observed, which is likely due to the distribution of vibrational states in ZnSeS QDs, which is a common feature for ternary nanocrystals [26]. The PL emission shows a signal located at ca. 395 nm belonging to the ZnSeS excitonic radiative recombination associated to a broad signal centered at ca. 480 nm corresponding to the defect-related emission emanating from ZnSeS nanocrystals. The band-edge PL is weak for QDs prepared with Se/S ratios of 25/75 and 50/50 but much more intense for the dots prepared with a Se/S ratio of 75/25. ZnSeS QDs prepared with an Se/S ratio of 50/50 and exhibiting an almost pure and strong defect-related emission were further used in this study.

The synthesis evolution of ZnSeS QDs was monitored by UV-visible and PL emission spectroscopy (Figure S2). After the injection of Se^2−^ and S^2−^ at room temperature, an absorption signal at ca. 350 nm and a PL emission at 480 nm could immediately be observed. The absorption signal slightly red-shifted upon prolonging the reaction time at 100 °C, indicating the growth of the ZnSeS nanocrystals. The PL emission wavelength is not affected by the reaction time, but the PL intensity gradually increased during the first 4 h and then slightly decreased, which was likely due to the introduction of defects during extended heating. A reaction time of 4 h was selected in further experiments.

Finally, we investigated the influence of the pH on the optical properties of ZnSeS QDs (Figure S3). No significant changes on the band-edge emission and on the trap emission were observed when the pH of the precursor solution was varied from 7 to 11. However, both the was more pronounced and the PL intensity was the highest when conducting the reaction at pH 11, likely due to the higher solubility of Zn-3-MPA complexes at basic pH. The pH value was fixed at 11 in latter experiments.

**Figure 1:**
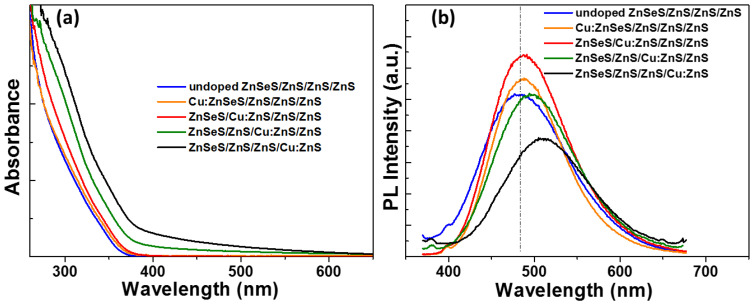
(**a**) UV-visible absorption and (**b**) PL emission spectra when varying the location of the Cu dopant in the dots (a loading of 2.5% in Cu was used).

The Cu-doping of ZnSeS QDs and the influence of the dopant location were next investigated (Figure 1). Upon doping of the ZnSeS core with 2.5% Cu, a shift of the PL emission from 480 to 486 nm and an increase of the PL QY from 10% to 13% were observed, indicating that Cu was introduced in the dots. However, Cu:ZnSeS QDs suffer from a modest photostability, likely due to the oxidation of Cu^+^ under irradiation in the presence of oxygen. This led us to investigate the doping in the ZnS shell deposited at the surface of the ZnSeS core. Using this strategy, a significant increase of the PL QY to 17% was reached. It is noteworthy that no significant shift of the PL emission was observed by doping Cu in the ZnSeS core or in the ZnS shell, suggesting that the energy level of Cu was not modified by this change of the local environment. A significant PL redshift was observed when Cu was introduced in the second or in the third ZnS monolayer covering the ZnSeS core (496 and 509 nm, respectively), but the PL intensity decreased as shown in Figure 1b, likely due to the weaker protection of Cu luminescent centers by the thinner ZnS shell.

**Figure 2:**
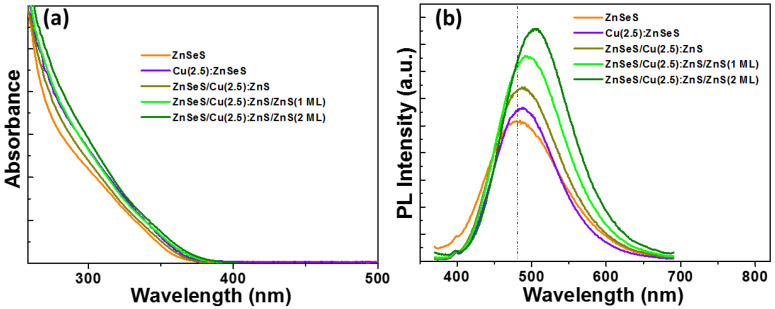
(**a**) UV-visible absorption and (**b**) PL emission spectra of ZnSeS, Cu(2.5):ZnSeS, ZnSeS/Cu(2.5):ZnS and ZnSeS/Cu(2.5):ZnS/ZnS QDs.

As previously indicated, the PL QY of ZnSeS/Cu:ZnS QDs is of 17%. The PL QY of the dots could further be increased to 21% and 25% by depositing 1 and 2 monolayers (ML) of ZnS at the periphery of ZnSeS/Cu:ZnS QDs, respectively, indicating that ZnS shells passivate the surface of ZnSeS/Cu:ZnS QDs without introducing further defects. This shelling was accompanied by a gradual shift of the PL emission from 488 nm for ZnSeS/Cu:ZnS QDs to 495 and 505 nm for ZnSeS/Cu:ZnS/ZnS (1 ML) and ZnSeS/Cu:ZnS/ZnS (2 ML), respectively, and by a slight shift of the UV-visible absorption edge, which further confirms the increase in size of the nanocrystals (Figure 2). The PL QY slightly decreased by further increasing the shell thickness, likely due to strain-induced interfacial defect sites caused by the lattice mismatch between the ZnSeS core and the ZnS shell.

**Figure 3:**
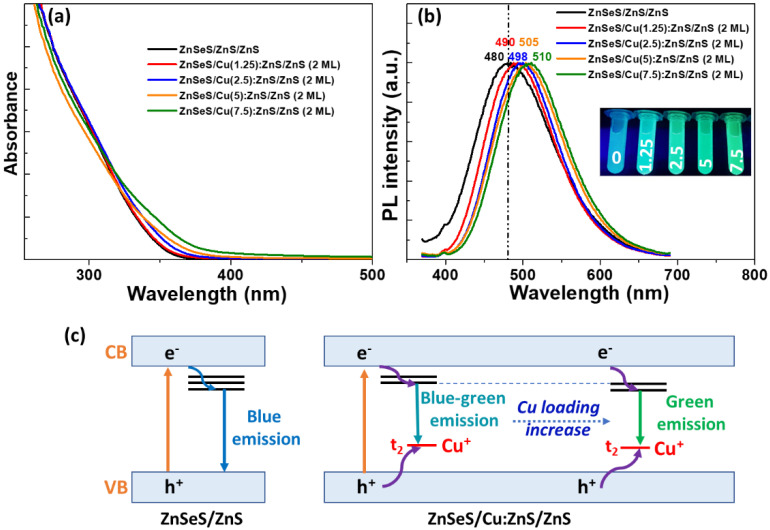
(**a**) UV-visible absorption and (**b**) normalized PL emission spectra of ZnSeS/Cu:ZnS/ZnS QDs when varying the dopant concentration from 1.25 to 7.5 mol%. The inset of (**b**) is a photograph taken under UV illumination of aqueous dispersions of the dots. (**c**) Schematic representation of PL emission mechanisms of ZnSeS/ZnS and ZnSeS/Cu:ZnS QDs and of the shift from blue-green to green emission when increasing the Cu loading.

The dopant loading was also found to play a key role for tailoring the PL properties of ZnSeS/Cu:ZnS/ZnS QDs. Figure 3a,b shows the UV-visible and the PL emission spectra of the dots when varying the Cu loading (1.25%, 2.5%, 5% and 7.5%). As the concentration of Cu increases, the absorption tail shifts towards higher wavelengths due to the narrowed bandgap associated with the Cu level located within the bandgap. Simultaneously, the PL emission shifts from 490 nm (1.25% Cu) to 510 nm (7.5% Cu). A photograph taken under UV light irradiation confirms the greener emission with the increase of the Cu loading (inset of Figure 3b). The shift of the Cu-related emission to higher wavelengths is consistent with previous reports [21,27]. It is likely that the substitutional defects generated by the Cu doping induce both an asymmetric atomic arrangement and an electron cloud distribution and thus an asymmetric crystal field. The density and the energy level of these defects likely depend on the Cu-loading and, based on the results described in Figure 3a,b, we can assume that a decrease in defect-related energy levels occurs when the dopant concentration increases (Figure 3c). These defects may trap electrons after their photo-excitation from the valence band to the conduction band. The recombination of these defects located at a lower energy level when the Cu load increases induces a shift of the PL emission towards longer wavelengths, as observed in Figure 3b.

The PL intensity is an increasing function of the Cu concentration up to 2.5% due to the incorporation of Cu luminescent centers in the nanocrystals. The highest PL QY (25%) was measured for the sample doped with 2.5% Cu, suggesting that this dopant concentration favors the transfer of photogenerated electrons in the defect states to the t_2_ level. By further increasing the Cu loading, a decrease of the PL intensity was observed, likely due to a concentration quenching effect originating from non-radiative energy transfers between neighboring Cu^+^ ions.

**Figure 4:**
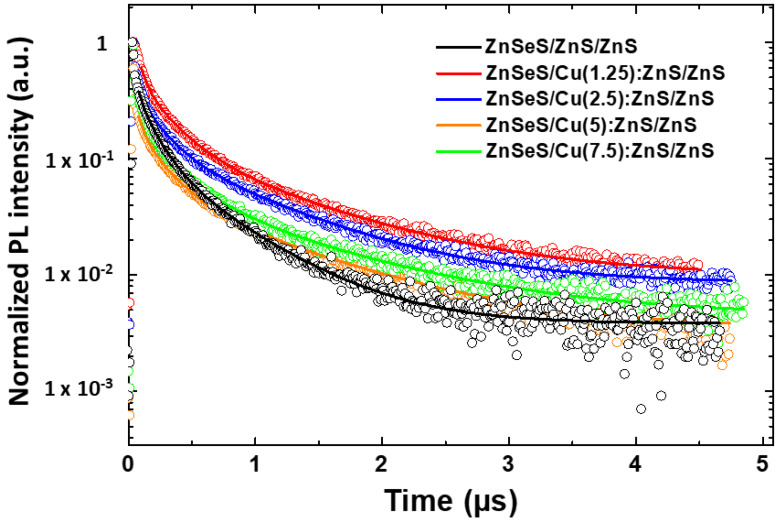
Normalized PL decay curves of ZnSeS/Cu:ZnS/ZnS (2 ML) QDs when varying the Cu dopant concentration. PL decay curves were measured at the maximum of the PL emission signals (see Figure 3b).

The PL decay kinetics of ZnSeS/Cu:ZnS/ZnS QDs when increasing the concentration of Cu from 1.25% to 7.5% were recorded to obtain information on the emission mechanism (Figure 4). The PL decay curves were fitted using a tri-exponential function using the equation *I*(t) = A_1_ exp (-t/*τ*_1_) + A_2_ exp (-t/*τ*_2_) + A_3_ exp (-t/*τ*_3_), where *τ*_1_, *τ*_2_ and *τ*_3_ are the time constants and A_1_, A_2_ and A_3_ the amplitudes of the components. The average lifetime τ_av_ was calculated using the formula *τ*_av_ = (A_1_*τ*_1_ + A_2_*τ*_2_ + A_3_*τ*_3_)/(A_1_ + A_2_ + A_3_), and all parameters are given in Table 1. Due to the presence of additional energy Cu *d*-states, the excited state lifetime of ZnSeS/Cu:ZnS/ZnS QDs (ca. 0.29 μs) is longer than both that of the undoped QDs (0.22 μs) and of core/shell Cu-doped ZnSeS/ZnS QDs doped in the core (0.164 μs) previously described [21]. The average PL lifetime of ZnSeS/Cu:ZnS/ZnS QDs of ca. 0.29 μs agrees well with those determined for Cu-doped QDs and indicates that the PL emission mostly originates from the Cu dopant-related transition and not from excitonic recombination or from defect states [28,29]. The short lifetime (τ_1_) corresponds to surface defect states, and the two longer PL decays (τ_2_ and τ_3_) may be associated either to the Cu-related emission or to the intrinsic defects (electron transition from donor to acceptor states like Zn vacancies and S or Se dangling bonds) located within the bandgap as previously described for Cu-doped ZnS or ZnSe nanocrystals [30,31]. For QDs doped with 1.25% of Cu, the long decay lifetimes are slightly higher than for other Cu doping content, but the contribution of surface defects is also marked for these nanocrystals, which may explain the lower PL QY (21%) of the QDs compared to the sample doped with 2.5% Cu (PL QY of 25%). For QDs doped with 2.5% Cu, the PL contribution related to the τ_3_ component is high compared to the other two components, which could suggest that τ_3_ corresponds to the Cu-related transition whereas τ_2_ to the donor-acceptor transition. The τ_3_ values slightly increase for the samples doped with 5% and 7.5% Cu, but the relative contributions markedly decrease compared to the dots doped with 2.5% Cu, which may explain the decrease of PL QYs observed for these samples (17% and 13% for QDs doped with 5% and 7.5% Cu, respectively).

### 3.2. Structural Characterizations and Photostability

The actual concentration of the Cu dopant was determined by ICP-OES analysis. For loadings in Cu of 1.25%, 2.5%, 5% and 7.5%, the actual concentrations after synthesis and purification were found to be 0.37%, 0.77%, 1.70% and 3.06%, respectively, indicating that Cu^+^ ions are difficult to insert into the crystalline network of the first ZnS shell.

**Figure 5:**
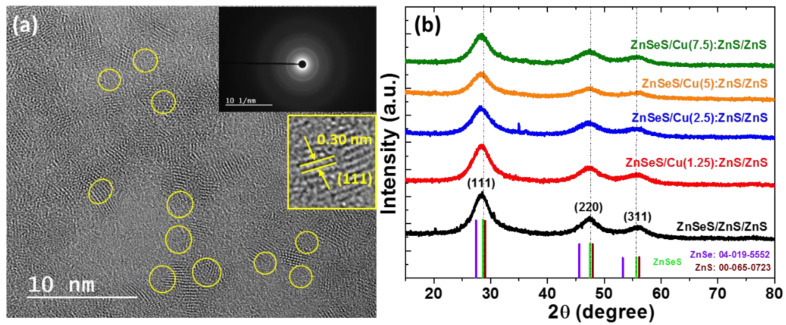
(**a**) HR-TEM image of ZnSeS/Cu(2.5):ZnS/ZnS QDs and (**b**) XRD patterns of ZnSeS/ZnS/ZnS and ZnSeS/Cu:ZnS/ZnS QDs. The insets of (**a**) are the SAED pattern and a magnification of the HR-TEM image.

A representative TEM image of the dots doped with 2.5% Cu is shown in Figure 5a, along with the SAED pattern and a HR-TEM image as the insets. ZnSeS/Cu(2.5):ZnS/ZnS (2 ML) QDs exhibit a nearly spherical morphology, and their average diameter is 2.1 ± 0.3 nm (see Figure S4 for the size distribution). The lattice spacing measured is 0.31 nm, in accordance with the (111) diffraction plane of cubic zinc blende ZnSe (0.327 nm) and ZnS (0.312 nm). The SAED pattern shows three concentric rings that can be assigned to the (111), (220) and (311) reflecting planes of the cubic zinc blende structure.

XRD patterns recorded when varying the dopant concentration confirm that the dots have a zinc blende structure (see JCPSC No 04-021-6302 for zinc sulfide selenide with a Se/S stoichiometry of 1/1) and that S^2−^ and Se^2−^ are well incorporated in the ZnSeS core (Figure 5b). The broad diffraction peaks further indicate the nanocrystalline nature of the samples. The diffraction peaks are located between those of cubic zinc blende ZnS and ZnSe, further confirming the successful preparation of the alloyed ZnSeS core. Although the ionic radius of Cu^+^ (0.06 nm) is smaller than that of Zn^2+^ (0.074 nm), the introduction of Cu into the dots only slightly affects their crystalline structure, and only a weak increase of the lattice parameter from 0.5435 to 0.5454 nm was detected when the dopant loading was increased from 0 to 7.5%.

**Figure 6:**
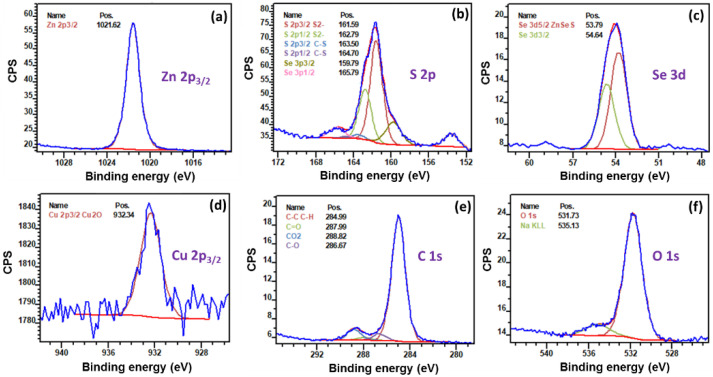
HR XPS spectra of (**a**) Zn 2p, (**b**) S 2p, (**c**) Se 3d, (**d**) Cu 2p, (**e**) C 1s and (**f**) O 1s.

XPS was further used to investigate the composition and the oxidation states of the elements in ZnSeS/Cu:ZnS/ZnS QDs. The XPS survey spectrum confirms the presence of Zn, Se, S, Cu, C, O and Na elements in the dots (Figure S5). The binding energy of Zn 2p_3/2_ is located at 1021.62 eV, which is typical of Zn^2+^ (Figure 6a). For S, the signals at 161.59 and 162.79 eV correspond to S 2p_3/2_ and S 2p_1/2_ of S^2−^ in the ZnSeS/ZnS crystal lattice, while the peaks at 163.50 and 164.70 eV correspond to S 2p_3/2_ and S 2p_1/2_ of the 3-MPA capping ligand, which indicate that S is in the −2 oxidation state (Figure 6b). XPS peaks observed at 53.79 and 54.64 eV can be attributed to Se 3d_5/2_ and Se 3d_3/2_ and confirm that Se is in the −2 oxidation state (Figure 6c). The small shifts in the peak position of Zn, S and Se towards higher binding energies compared to the literature likely originate from interactions with the Cu^+^ dopant. For Cu, one signal at 932.34 eV corresponding to the 2p_3/2_ of Cu^+^ can be observed [32], indicating that Cu^2+^ is reduced into Cu^+^ by Na_2_S or NaHSe during the synthesis of the dots (Figure 6d). The signals observed for C 1s at 284.99, 287.99, 288.82 and 286.67 eV correspond to C-C, C-H, C-O and C=O bonds, respectively, in the 3-MPA ligand (Figure 6e). Finally, the O 1s signal is observed at 531.73 eV (Figure 6f).

The reduction of Cu^2+^ into Cu^+^ was confirmed by ESR (Figure S6). The ESR spectra of the QDs doped with 5% or 7.5% Cu show a very weak signature around g = 2 (3350 G). Although this clearly originates from traces of Cu^2+^ [33], the intensity is much too small compared to the content of Cu and is thus clearly in agreement with most of the Cu^2+^ having been reduced into Cu^+^ during the synthesis. The poorly resolved signal also suggests that several different environments exist around remaining Cu^2+^ ions.

**Figure 7:**
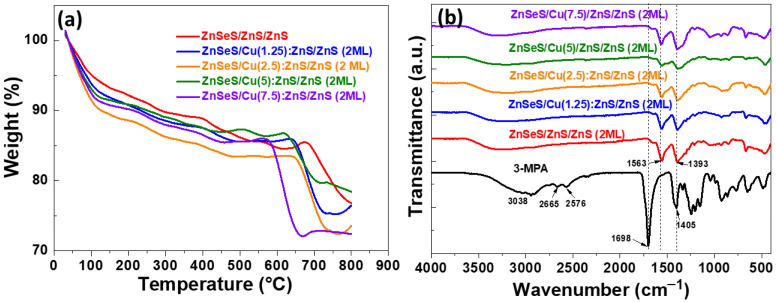
(**a**) TGA curves and (**b**) FT-IR spectra of 3-MPA and of undoped and ZnSeS/Cu:ZnS/ZnS QDs.

Thermogravimetric analyses (TGAs) of ZnSeS/Cu:ZnS/ZnS QDs show a gradual weight loss of ca. 15% between 100 and 550 °C originating from the removal of chemisorbed water molecules and from the decomposition of the 3-MPA ligand (Figure 7a). The second weight loss observed above 600 °C could be assigned to the partial decomposition of the inorganic core. These results show that a relatively thick shell of ligand covers ZnSeS/Cu:ZnS/ZnS QDs and ensures their good dispersibility in aqueous media (*vide infra*).

The capping of the QDs by the 3-MPA ligand was further confirmed by FT-IR (Figure 7b). Pure 3-MPA exhibits a broad signal at ca. 3038 cm^−1^ (O-H stretching), two peaks at 2665 and 2576 cm^−1^ (S-H stretchings) and signals at 1698 and 1405 cm^−1^ (asymmetric and symmetric stretching modes of the C=O function, respectively). The S-H stretchings disappear in QDs spectra, which confirms that 3-MPA is linked to the surface Zn atoms via the thiol function. The asymmetric and symmetric vibrations of the carboxylate CO_2_^−^ group can be observed at 1563 and 1393 cm^−1^, respectively, for ZnSeS/Cu:ZnS/ZnS QDs.

**Figure 8:**
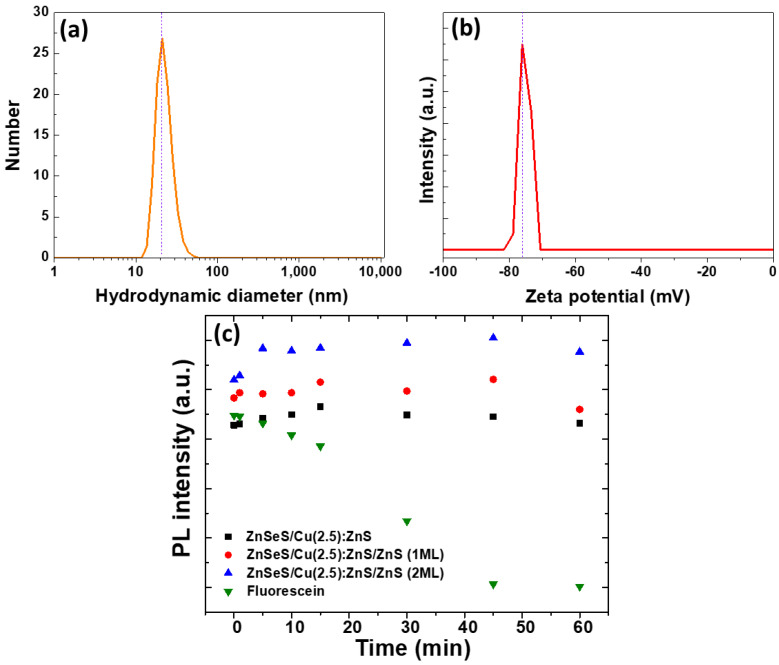
(**a**) DLS hydrodynamic diameter in number and (**b**) Zeta potential of ZnSeS/Cu:ZnS/ZnS QDs. (**c**) Photostability of ZnSeS/Cu(2.5):ZnS, ZnSeS/Cu(2.5):ZnS/ZnS QDs and of fluorescein under the continuous illumination of an Hg–Xe lamp (irradiance of 50 mW/cm^2^).

DLS was used to determine the size distribution profile of ZnSeS/Cu:ZnS/ZnS QDs in aqueous solution. A representative DLS analysis of the dots doped with 2.5% Cu shows that their average hydrodynamic diameter is ca. 20 ± 2.5 nm (Figure 8a). This value is significantly higher than that of the inorganic core determined by TEM (2.1 ± 0.3 nm) and suggests that QDs assemble into clusters of a few nanocrystals in solution despite their negative Zeta potential (−76 ± 4 mV) (Figure 8b).

ZnSeS/Cu:ZnS/ZnS QDs show no tendency to aggregate after dispersion at high concentration in aqueous solution, and no alteration of their optical properties was noticed. In dilute solution, the irreversible detachment of the 3-MPA ligand originating from the impaired dynamic equilibrium of detachment and rebinding of 3-MPA favors the agglomeration of the dots due to insufficient repulsive electrostatic forces, and a decrease of their PL QY is observed [34].

We also evaluated the photostability of ZnSeS/Cu(2.5):ZnS and ZnSeS/Cu(2.5):ZnS/ZnS QDs and of fluorescein upon illumination with an Hg–Xe lamp (intensity of 50 mW/cm^2^ at the surface of the QDs or fluorescein aqueous solution) and in the presence of oxygen. As can be seen in Figure 8c, the nanocrystals remain well photoluminescent during the 60 min of irradiation (a slight decrease of the PL intensity can only be observed after 75 min of irradiation). The photo-enhancement (by a factor of 5–15%) observed during the first 10 min of irradiation likely originates from the partial decomposition of the 3-MPA ligand followed by the association of S^2−^ anions released with Zn^2+^ surface atoms to create a ZnS extra shell that further improves the optical properties of the dots. No shift was observed in the UV-visible absorption and PL emission spectra of the QDs, indicating that the Cu dopant was well protected from photo-oxidation (Figures S7 and S8). Under the same illumination conditions, fluorescein is fully bleached in 45 min, which shows the interest of ZnSeS/Cu:ZnS/ZnS QDs compared to organic dyes for applications in which long-term PL stability and high brightness are required.

## 4. Conclusions

In summary, a mild aqueous phase synthesis of high quality core/shell/shell ZnSeS/Cu:ZnS/ZnS QDs was developed. ZnSeS/Cu:ZnS/ZnS QDs exhibit an average diameter of ca. 2.1 ± 0.3 nm and a good crystallinity. The highest PL QY (25%) was obtained by doping Cu in the first ZnS shell covering the alloyed ZnSeS core, and the nanocrystals were demonstrated to be photostable. Finally, the PL emission can be tuned from 480 to 510 nm by increasing the Cu loading from 0 to 7.5%. Due to their stable PL emission in the blue-green region of the visible spectrum and to their good colloidal stability, ZnSeS/Cu:ZnS/ZnS QDs should be of interest for applications such as bio-imaging or sensing.

## Figures and Tables

**Table 1:** PL decay lifetimes of ZnSeS/Cu:ZnS/ZnS when varying the Cu loading from 0% to 7.5%.

Cu doping (%)	A_1_	τ_1_ (µs)	A_2_	τ_2_ (µs)	A_3_	τ_3_ (µs)	τ_a_ (µs)
0	0.082	0.033	0.232	0.143	0.099	0.559	0.222
1.25	0.268	0.054	0.304	0.372	0.130	0.968	0.302
2.5	0.173	0.03	0.219	0.188	0.119	0.842	0.291
5	0.107	0.046	0.126	0.212	0.050	0.973	0.284
7.5	0.157	0.062	0.125	0.062	0.051	1.077	0.295

## Data Availability

Not applicable.

## Supplementary Materials

The following supporting information can be downloaded at https://www.mdpi.com/article/10.3390/nano12183254/s1, Figure S1. (a) UV-visible absorption and (b) PL emission spectra of ZnSeS QDs when varying the Se/S molar ratio. The inset of (b) is a photograph taken under UV illumination of aqueous dispersions of the dots. Figure S2. (a) UV-visible absorption and (b) PL emission spectra at different reaction stages. The inset of (b) is a photograph taken under UV illumination of aqueous dispersions of the dots. Figure S3. (a) UV-visible absorption and (b) PL emission spectra of ZnSeS QDs when varying the pH of the reaction. Reactions were conducted for 4 h using a Se/S ratio of 50/50. Figure S4. Particle size distribution expressed in number of ZnSeS/Cu(2.5):ZnS/ZnS (2 ML) QDs determined by TEM. Figure S5. XPS overview spectrum of ZnSeS/Cu(2.5):ZnS/ZnS QDs. Figure S6. EPR spectra of ZnSeS/Cu(5 and 7.5):ZnS/ZnS QDs recorded at room temperature. Figure S7. Evolution of UV-visible absorption spectra of (a) ZnSeS/Cu(2.5):ZnS, (b-c) of ZnSeS/Cu(2.5):ZnS/ZnS QDs and of (d) fluorescein during the continuous irradiation of a Hg/Xe lamp. Figure S8. Evolution of PL emission spectra of (a) ZnSeS/Cu(2.5):ZnS, (b-c) of ZnSeS/Cu(2.5):ZnS/ZnS QDs and of (d) fluorescein during the continuous irradiation of a Hg/Xe lamp.
