# Supplementary material for: Highly Luminescent and Photostable Core/Shell/Shell ZnSeS/Cu:ZnS/ZnS Quantum Dots Prepared via a Mild Aqueous Route
Source: Nanomaterials (Basel). 2022 Sep 19;12(18):3254. doi: 10.3390/nano12183254 (PMC9504198; doi:10.3390/nano12183254)
Supplement: Supplementary file 1 [file nanomaterials-12-03254-s001.zip › nanomaterials-1894258-supplementary.pdf]

Supporting Information

Article

Highly Luminescent and Photostable Core/Shell/Shell ZnSeS/Cu:ZnS/ZnS Quantum Dots Prepared via a Mild Aqueous Route

Salima Mabrouk [1,2], Hervé Rinnert [3], Lavinia Balan [4], Jordane Jasniewski [5], Sébastien Blanchard [6], Ghouti Medjahdi [3], Rafik Ben Chaabane [2] and Raphaël Schneider [1,*]

1 Université de Lorraine, CNRS, LRGP, F-54000 Nancy, France
2 Laboratoire Interfaces et Matériaux Avancés, LIMA, LR011ES55, Faculté des Sciences de Monastir, Avenue de l'Environnement, Monastir 5019, Tunisia
3 Université de Lorraine, CNRS, IJL, F-54000 Nancy, France
4 CEMHTI-UPR 3079 CNRS, Site Haute Température, 1D Avenue de la Recherche Scientifique, 45071 Orléans, France
5 Université de Lorraine, LIBio, F-54000 Nancy, France
6 Sorbonne Université, CNRS, Institut Parisien de Chimie Moléculaire, IPCM, F-75005 Paris, France
* Correspondence: raphael.schneider@univ-lorraine.fr; Tel.: +33-372743790

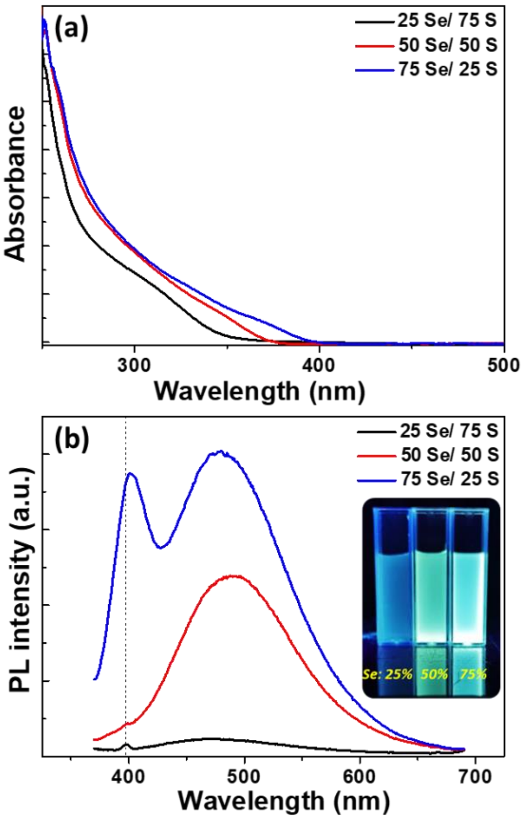

Figure S1. (a) UV-visible absorption and (b) PL emission spectra of ZnSeS QDs when varying the Se/S molar ratio. The inset of (b) is a photograph taken under UV illumination of aqueous dispersions of the dots.

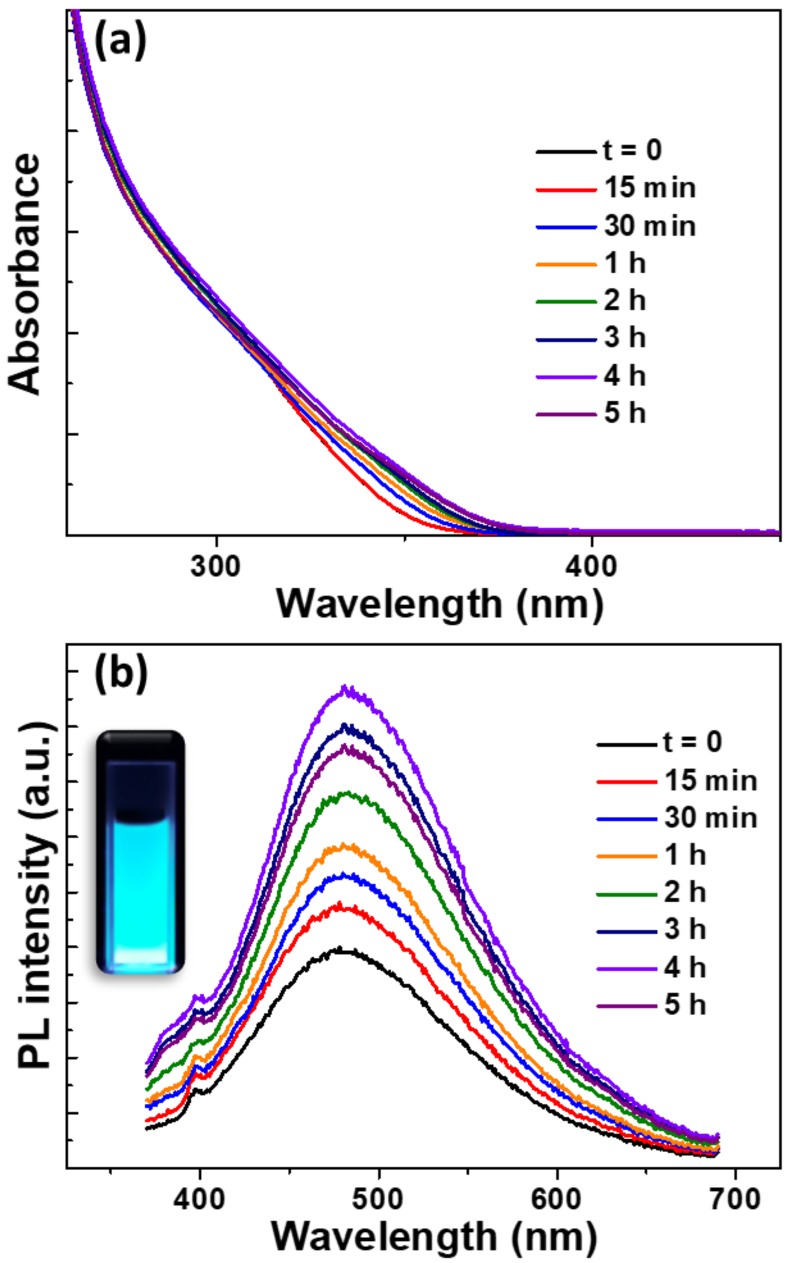

Figure S2. (a) UV-visible absorption and (b) PL emission spectra at different reaction stages. The inset of (b) is a photograph taken under UV illumination of aqueous dispersions of the dots.

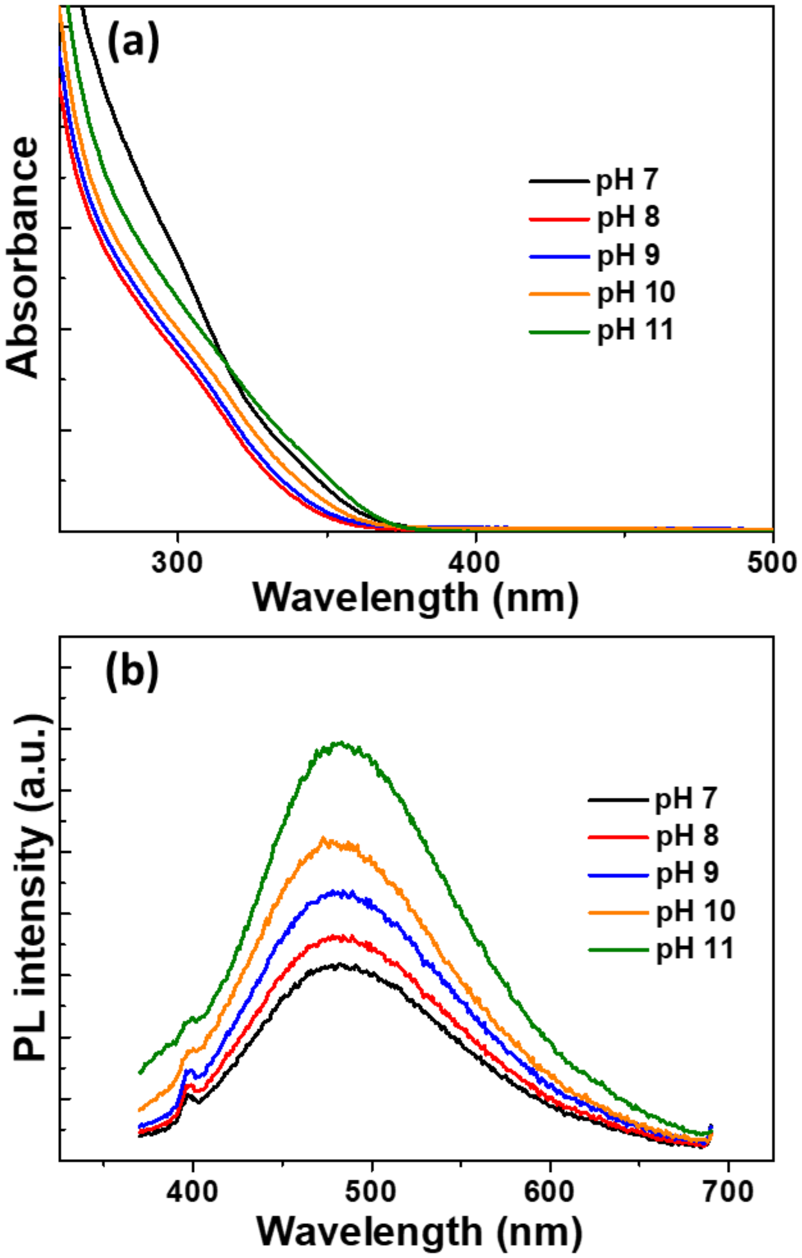

Figure S3. (a) UV-visible absorption and (b) PL emission spectra of ZnSeS QDs when varying the pH of the reaction. Reactions were conducted for 4 h using a Se/S ratio of 50/50.

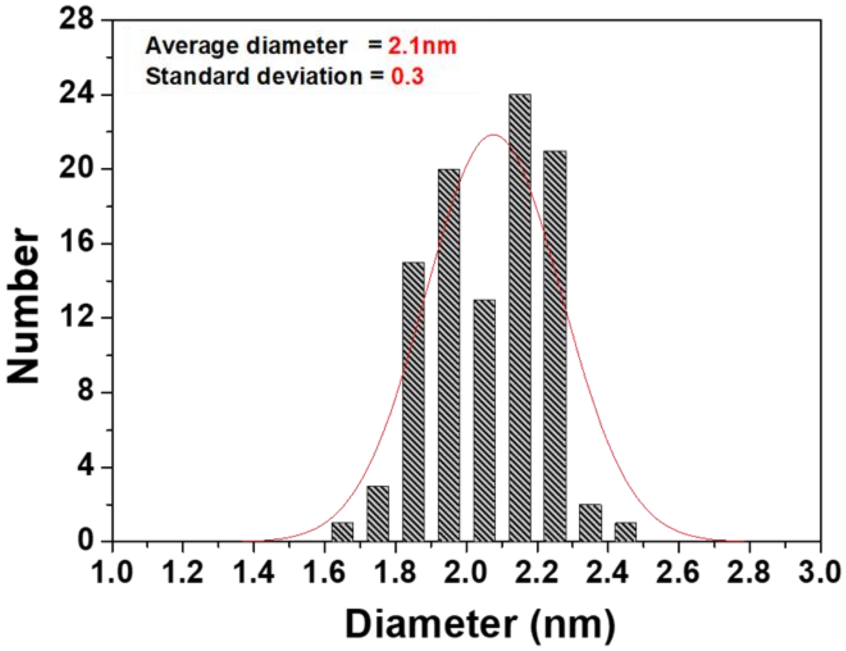

Figure S4. Particle size distribution expressed in number of ZnSeS/Cu(2.5):ZnS/ZnS (2ML) QDs determined by TEM.

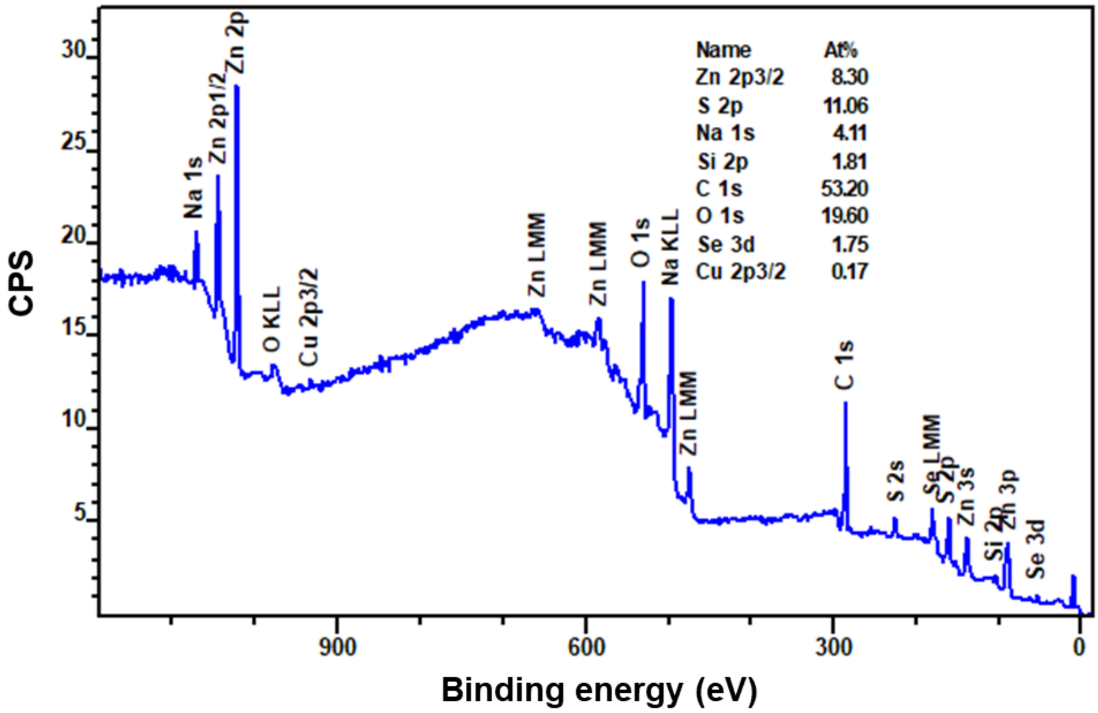

Figure S5. XPS overview spectrum of ZnSeS/Cu(2.5):ZnS/ZnS QDs.

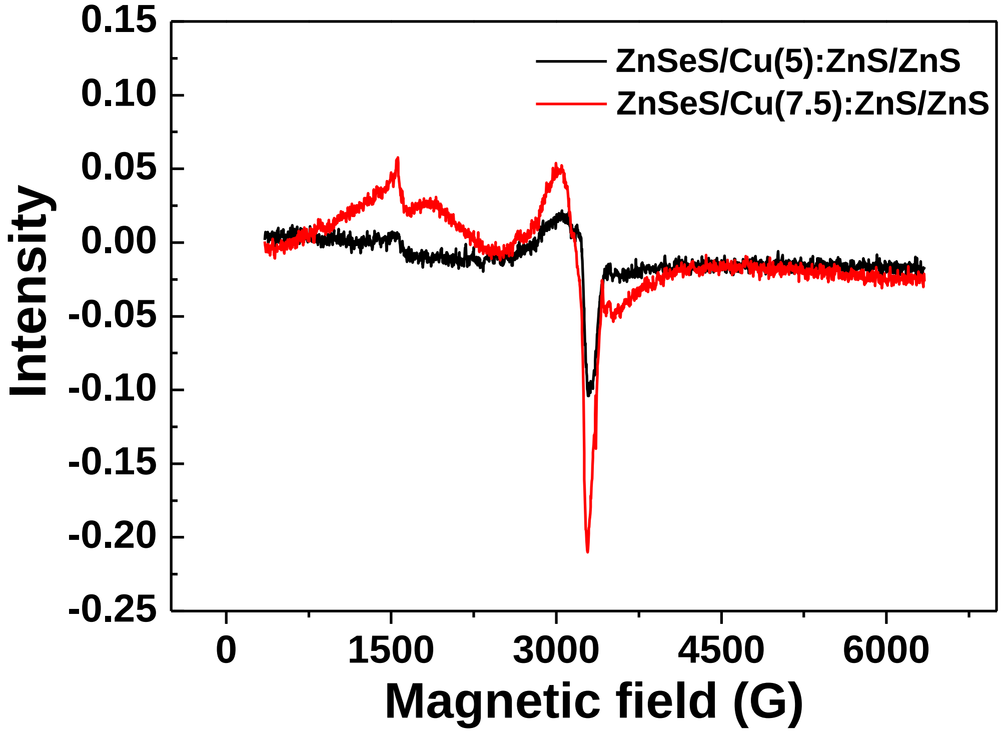

Figure S6. EPR spectra of ZnSeS/Cu(5 and 7.5):ZnS/ZnS QDs recorded at room temperature.

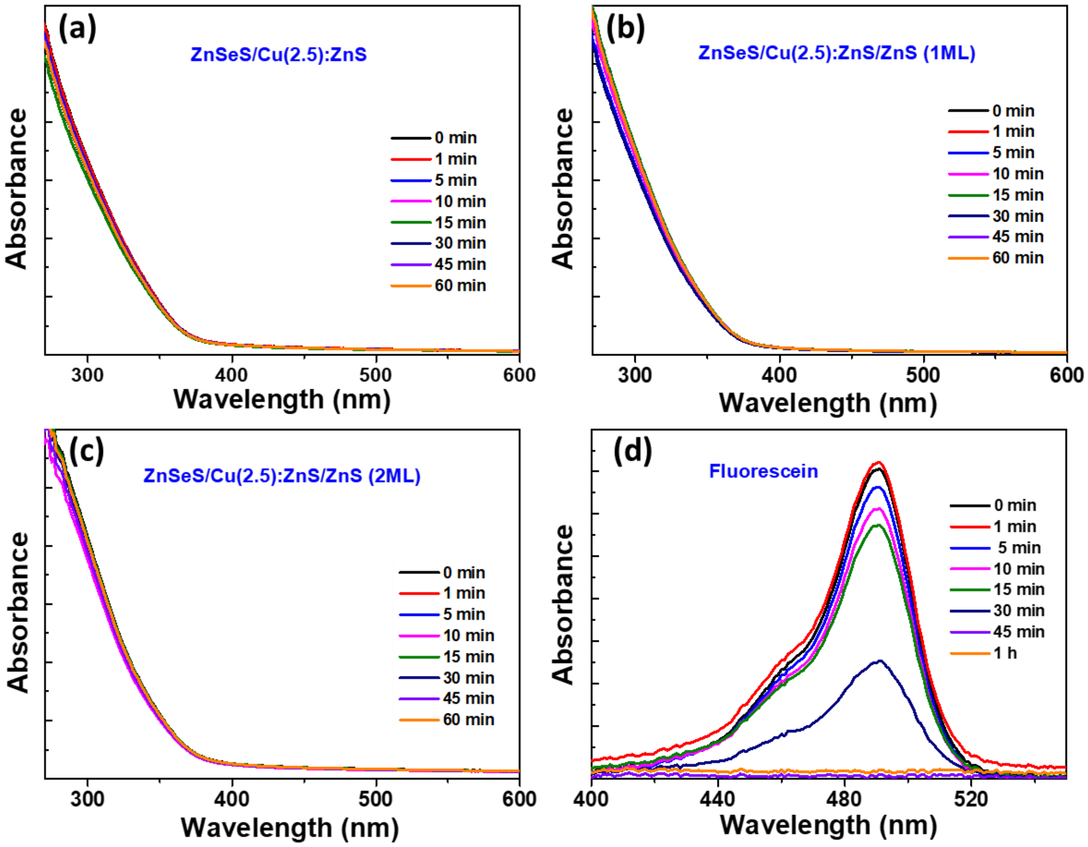

Figure S7. Evolution of UV-visible absorption spectra of (a) ZnSeS/Cu(2.5):ZnS, (b-c) of ZnSeS/Cu(2.5):ZnS/ZnS QDs and of (d) fluorescein during the continuous irradiation of a Hg/Xe lamp.

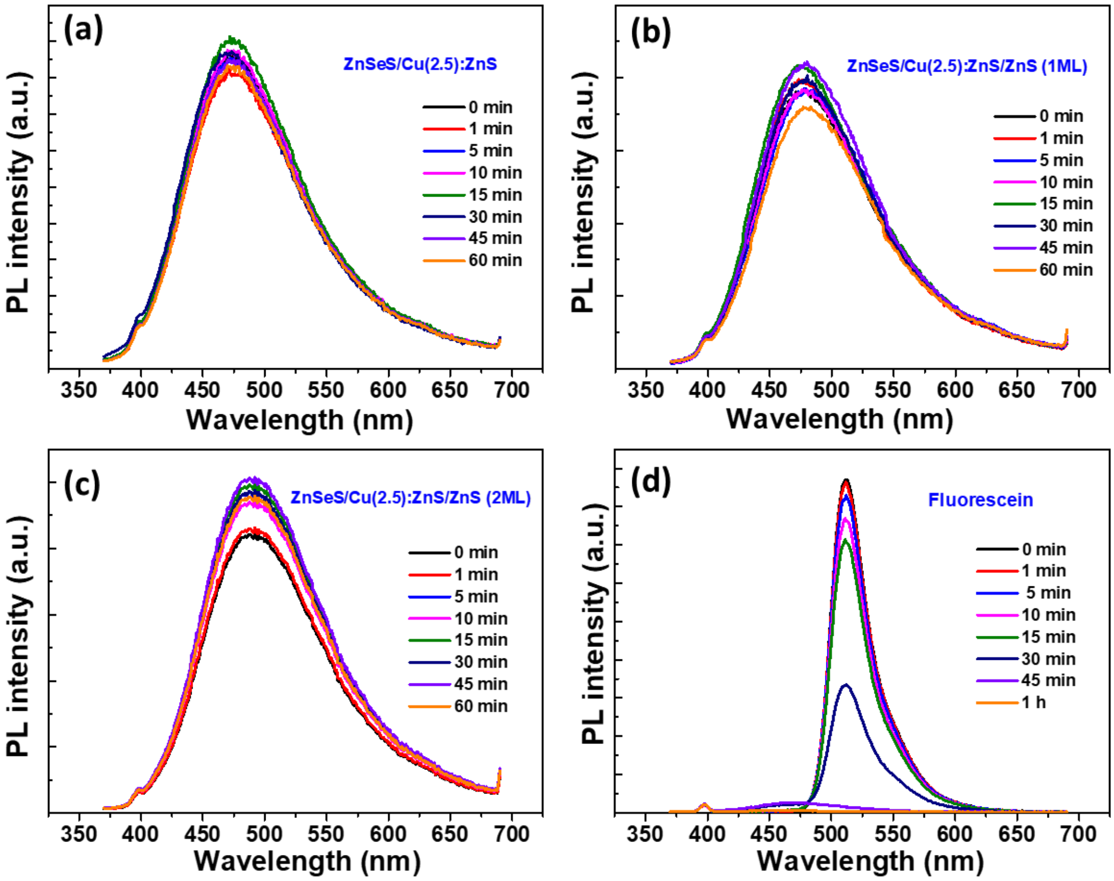

Figure S8. Evolution of PL emission spectra of (a) ZnSeS/Cu(2.5):ZnS, (b-c) of ZnSeS/Cu(2.5):ZnS/ZnS QDs and of (d) fluorescein during the continuous irradiation of a Hg/Xe lamp.
